# Impact of the *Superoxide Dismutase 2* Val16Ala Polymorphism on the Relationship between Valproic Acid Exposure and Elevation of γ-Glutamyltransferase in Patients with Epilepsy: A Population Pharmacokinetic-Pharmacodynamic Analysis

**DOI:** 10.1371/journal.pone.0111066

**Published:** 2014-11-05

**Authors:** Naoki Ogusu, Junji Saruwatari, Hiroo Nakashima, Madoka Noai, Miki Nishimura, Mariko Deguchi, Kentaro Oniki, Norio Yasui-Furukori, Sunao Kaneko, Takateru Ishitsu, Kazuko Nakagaswa

**Affiliations:** 1 Division of Pharmacology and Therapeutics, Graduate School of Pharmaceutical Sciences, Kumamoto University, Kumamoto, Japan; 2 Department of Neuropsychiatry, Hirosaki University School of Medicine, Hirosaki, Japan; 3 Kumamoto Saishunso National Hospital, Kumamoto, Japan; 4 Kumamoto Ezuko Ryoiku Iryo Center, Kumamoto, Japan; 5 Center for Clinical Pharmaceutical Sciences, Kumamoto University, Kumamoto, Japan; CNRS UMR7275, France

## Abstract

**Background:**

There has been accumulating evidence that there are associations among γ-glutamyltransferase (γ-GT) elevation and all-cause mortality, cardiovascular diseases and metabolic diseases, including nonalcoholic fatty liver disease. The primary objective of this study was to evaluate the impact of the most common and potentially functional polymorphisms of antioxidant enzyme genes, i.e. *superoxide dismutase 2 (SOD2)*, *glutathione S-transferase M1* and *glutathione S-transferase T1*, on the γ-GT elevation during valproic acid (VPA) therapy.

**Methods and Findings:**

This retrospective study included 237 and 169 VPA-treated Japanese patients with epilepsy for population pharmacokinetic and pharmacokinetic-pharmacodynamic analyses, respectively. A nonlinear mixed-effect model represented the pharmacokinetics of VPA and the relationships between VPA exposure and γ-GT elevation. A one-compartment model of the pharmacokinetic parameters of VPA adequately described the data; while the model for the probability of the γ-GT elevation was fitted using a logistic regression model, in which the logit function of the probability was a linear function of VPA exposure. The *SOD2* Val16Ala polymorphism and complication with intellectual disability were found to be significant covariates influencing the intercept of the logit function for the probability of an elevated γ-GT level. The predicted mean percentages of the subjects with γ-GT elevation were about 2- to 3-fold, 3- to 4-fold and 4- to 8-fold greater in patients with the *SOD2* Val/Val genotype but without any intellectual disability, those with the *SOD2* Val/Ala or Ala/Ala genotype and intellectual disability and those with the *SOD2* Val/Val genotype and intellectual disability, respectively, compared to those with the *SOD2* Val/Ala or Ala/Ala genotype without intellectual disability.

**Conclusion:**

Our results showed that the *SOD2* Val16Ala polymorphism has an impact on the relationship between VPA exposure and γ-GT elevation in patients with epilepsy. These results suggest that determining the *SOD2* genotype could be helpful for preventing the VPA-induced γ-GT elevation.

## Introduction

γ-Glutamyltransferase (γ-GT) is a hepatic and biliary enzyme synthesized by hepatocytes as well as the epithelial cells of the intra-hepatic bile ducts [1]. Measurements of the γ-GT activity in the serum are used clinically as a liver function parameter [1], [2]. The serum γ-GT level is also a biomarker of excessive alcohol consumption [1], [2]. The available evidence indicates that an elevated γ-GT level is related to nonalcoholic fatty liver disease (NAFLD) [3], [4]. The oxidative stress and inflammation caused by NAFLD might contribute to the elevation of γ-GT [5], [6]. There is also strong evidence for associations between the γ-GT activity and all-cause mortality, cardiovascular disease, type 2 diabetes, metabolic syndrome, insulin resistance and obesity [2], [7], [8].

Valproic acid (VPA) is one of the most widely prescribed antiepileptic drugs worldwide [9]. VPA is also used to treat migraines and bipolar, mood, anxiety and psychiatric disorders [9]. Therapeutic drug monitoring is the measurement of the blood level of a drug to ensure that its concentration is within the therapeutic range [10]. Since the dose requirements for VPA are highly variable, therapeutic drug monitoring of VPA is commonly used [10]. Long-term treatment with VPA has been associated with metabolic and endocrine disorders, such as weight gain and hyperinsulinemia, which may contribute to the increased cardiovascular risk observed in patients with epilepsy [9]. Recently, NAFLD has emerged as a common chronic liver condition in VPA-treated patients [9], [11], [12].

Mitochondrial dysfunction has been implicated in the pathogenesis of VPA-induced hepatotoxicity [9], [13]. Superoxide dismutase 2 (SOD2, also known as manganese superoxide dismutase) plays a critical role in the detoxification of mitochondrial reactive oxygen species [13], [14]. The T to C nucleotide polymorphism (rs4880, Val16Ala) has been identified in exon 2 of the human *SOD2* gene [15]. The Ala variant is more efficiently imported into the mitochondria than the Val variant, thus resulting in increased mitochondrial SOD2 homotetramer activity derived from the Ala precursor variant [15]. Our previous case-control study demonstrated a possible association between the *SOD2* Val/Val genotype and the VPA-induced elevation of γ-GT [16].

The glutathione *S*-transferase (GST) supergene family consists of phase 2 detoxifying enzymes [17]. GST plays a crucial role in antioxidant defense mechanisms by detoxifying electrophilic xenobiotics and inactivating a variety of endogenous byproducts of oxidative stress [17]. The most extensively studied *GST* polymorphisms occur in two isozymes, i.e. GST mu 1 (GSTM1) and GST theta 1 (GSTT1) [17]. The most common polymorphism in the human *GSTM1* or *GSTT1* gene is a deletion of the whole gene (“null” genotype), which results in a lack of functional activity of the enzyme [17]. The two common deletion polymorphisms of *GSTM1* and *GSTT1* have been reported to be associated with an increased susceptibility to certain oxidative stress-related diseases [18]–[20]. In a previous case-control study, an association of the *GSTM1* null and *GSTT1* null genotypes with an increased γ-GT levels was reported in VPA-treated Japanese patients with epilepsy [21].

In this study, we applied population pharmacokinetic (PK)-pharmacodynamic (PD) modeling to describe the VPA-induced γ-GT elevation in patients with epilepsy. The primary objective of this retrospective study was to evaluate the impact of the most common and potentially functional polymorphisms in three antioxidant enzyme genes, i.e. *SOD2*, *GSTM1* and *GSTT1*, on the relationship between the VPA exposure and the risk of γ-GT elevation during long-term VPA therapy.

## Materials and Methods

### Participants and Study Design

This retrospective study was approved by the ethics committees of Kumamoto Saishunso National Hospital and the Faculty of Life Sciences, Kumamoto University (Kumamoto, Japan). This study included patients who were being treated at Kumamoto Saishunso National Hospital (Kumamoto, Japan) between June 1989 and April 2011. Four hundred and fifty-six Japanese patients with epilepsy and/or their parents gave their written informed consent to participate in this study. For the PK analysis, patients were included if they fulfilled all of the following conditions: had been receiving sustained-release VPA for three weeks or longer and were not taking any drugs that might alter the clearance of VPA, except for antiepileptic drugs; and having detailed medical data available. For the PK-PD analysis, patients were included if they fulfilled all of the criteria for the PK analysis, as well as the following conditions: a history of treatment with VPA for one year or longer; not taking any drugs that may affect the liver function, except for antiepileptic agents; and no history of either viral or alcoholic liver disease.

For all patients, the most appropriate antiepileptic drug was chosen according to the treatment guidelines of the Japan Epilepsy Society. The treatment was changed to another drug if the seizures remained uncontrolled, if drug-precipitated seizures were suspected or if the patient had any intolerable adverse drug reaction(s). At each follow-up visit, the clinical information was recorded. For all patients, VPA was initiated at a dose of 15–40 mg/kg/day for children (400–1,200 mg/day for adults) and was escalated at weekly intervals by 5–10 mg/kg/day (for children) or 200 mg/day (for adults) for each step up to the maximum tolerated dose.

The patients’ medical information was retrospectively obtained from their medical records. For every patient, the data included demographics, the VPA dose and schedule, diagnoses, seizure frequency and concomitant medications evaluated at every visit during the VPA therapy. The types of seizures and epileptic syndromes were classified according to the guidelines of the International League Against Epilepsy [22]. We used the International Classification of Diseases, 10th Revision (ICD-10) to identify individuals with moderate or severe intellectual disability (ICD-10 F70–F79, consistent with IQ scores <50), since intellectual disability is known to be common in patients with epilepsy [23]. Clinical laboratory tests, including serum γ-GT measurements, were performed regularly and when necessary. Blood samples were collected for routine therapeutic drug monitoring or to assess the patient for adverse events. Therefore, blood sampling was performed when necessary at the time of the visit for each patient, and thus, there were differences in the timing of blood collection among the patients (e.g., morning and afternoon). The data were retrospectively reviewed independently by a physician and by clinical pharmacists.

### Genotyping

Genomic DNA was isolated from EDTA blood samples using a DNA extractor WB kit (Wako Pure Chemical Industries, Ltd. Osaka, Japan). Null genotypes of *GSTM1* and *GSTT1* were determined using polymerase chain reaction (PCR) amplification based on the presence or absence of a PCR amplification product according to the previously described method [20]. The *GST* genotypes were classified as follows: subjects with homozygous deleted alleles (i.e. the “null” genotype) and others (i.e. the “present” genotype). The *SOD2* Val16Ala (c.47T>C; rs4880) polymorphism and the three most common polymorphisms of *cytochrome P450 (CYP) 2C9* and *CYP2C19* enzymes that are involved in VPA metabolism [24], i.e. CYP2C9*3 (c.1075A>C; rs1057910), CYP2C19*2 (c.681G>A; rs4244285) and CYP2C19*3 (c.636G>A; rs4986893), were genotyped using real-time PCR with 5′-nuclease allele discrimination assays (Step One Plus Real-Time PCR system version 2.1; Applied Biosystems, Tokyo, Japan). Genotyping for rs4880, rs1057910, rs4244285 and rs4986893 was performed using commercially available assays (assay IDs: C_8709053_10, C_27104892_10, C25986767_70 and C_27861809_10, respectively), according to the manufacturer’s protocol [25], [26]. All reagents were purchased from Applied Biosystems (Tokyo, Japan). Regarding *SOD2* and *CYP2C9*, the genotypes were classified as follows: homozygous for the wild-type allele (i.e. the *SOD2* Val allele or CYP2C9*1 allele), heterozygous for the wild-type and mutant alleles and homozygous for the mutant allele (i.e. the *SOD2* Ala allele or CYP2C9*3 allele). Regarding *CYP2C19*, the genotypes were classified into three groups: homozygous extensive metabolizers (EMs) (*1/*1 genotype), heterozygous EMs (*1/*2 or *1/*3 genotype) and poor metabolizers (PMs) (*2/*2, *3/*3 or *2/*3 genotype).

To ensure the quality of the genotyping, we included DNA samples as internal controls, hidden samples of known genotypes and a negative control (water). The genotype distributions of *SOD2* Val16Ala, CYP2C9*3, CYP2C19*2 and CYP2C19*3 were tested for Hardy-Weinberg equilibrium using the χ^2^ test. A *P* value <0.05 was considered to be statistically significant. The statistical analyses were performed using the R software program (version 3.0.0; R Foundation for Statistical Computing, Vienna, Austria).

### Assessment of the Serum VPA Concentrations

The serum VPA concentrations were evaluated using the steady-state therapeutic drug monitoring data from clinical practice using a homogenous enzyme immunoassay (Cobas Mira; Roche Diagnostics, Basel, Switzerland). The measurements were obtained in accordance with the manufacturer’s protocol. The within- and between-day coefficients of variation for determining VPA were <10% and the lower limit of quantification was 1 mg/L. Any therapeutic drug monitoring data obtained from patients with suspected temporary noncompliance were excluded from the analysis. The suspected temporary noncompliance was determined by assessing whether the serum concentrations were under the limit of quantification, as well as whether the medications were taken based on the pill counts.

### Assessment of the Serum γ-GT Levels

The γ-GT levels were measured using the standard method recommended by the Japan Society of Clinical Chemistry in daily practice at Kumamoto Saishunso National Hospital. Since the γ-GT level shows large inter-individual variability and it is correlated with age and sex [27], we defined an elevated γ-GT level as an increase over the upper limit of the normal range, which was stratified by age and sex [28]. For PK-PD modeling, we included all γ-GT levels measured after the start of VPA therapy in each patient, with the exception of the data collected in the early phase of VPA therapy (i.e. <6 months after the initiation of VPA therapy), in order to exclude any confounding effects due to transient γ-GT elevation after the commencement of VPA therapy [9], [29].

### Population PK and PK-PD Modeling

All analyses used during the population PK and PK-PD modeling were carried out with a nonlinear mixed-effect model (NONMEM, version 7.2.0; ICON Dev Soln, Ellicott City, MD). First-order conditional estimation with the ADVAN 2 TRANS 2 subroutine was used for PK parameter estimation. The ADVAN 2 subroutine contained the appropriate equations for a one-compartment model with first-order absorption and elimination, while the TRANS 2 subroutine parameterized the models in terms of the clearance and volume. The first-order conditional estimation with the use of laplacian estimation was used for the development of the PK-PD model.

### PK Modeling

A one-compartment model with first-order absorption and lag time was used to select the structural model for VPA. During model development, all compartment models were parameterized in terms of values of the absorption rate constant (*K*a), apparent oral clearance (*CL*/*F*), volume of distribution (*Vd/F*) and the absorption lag time (*ALAG*). A variance model was established by separately evaluating an additive error model, a proportional error model and an exponential error model for the inter-individual variability of each parameter and the variability of any residual errors. The inter-individual variability was best described using an exponential error model. The residual unexplained variability, which includes other unexplained factors of variability (e.g., model misspecification, assay errors), was best described using a proportional error model.

A regression model was developed using the forward-inclusion and backward-elimination methods. First, each covariate was incorporated nonlinearly into the basic regression model. The influence of covariates on the PK parameters was systematically tested in a forward-building model using different equations for continuous covariates (e.g., age) and categorical covariates (e.g., *CYP2C9* genotype) according to the previously described method [30]. From the basic model, important covariates were identified by plotting the estimates versus the covariates. The influence of these fixed effects was evaluated using the objective function. The full model was created by incorporating all covariates, which thus led to a significant decrease in the objective function. The objective function of the full model was used to test the effects of removing each covariate from the full model. Changes in the objective function of at least 3.84 [χ^2^, *P*<0.05; degree of freedom (df) = 1] and 5.99 (χ^2^, *P*<0.05; df = 2) were considered to be significant during the forward-inclusion and backward-elimination analyses. The covariates tested were the age, gender, body weight, daily VPA dose, *CYP2C9* genotype, *CYP2C19* genotype and co-administered antiepileptic drugs on the *CL/F* and body weight and the daily VPA dose on the *Vd*/*F*. At this step, typical values of the PK parameters were also considered.

### PK-PD Modeling

The final individual PK parameters, determined from the population PK model, were fixed in the PK-PD analysis. A binomial scale was used to represent the serum γ-GT elevation, with 0 indicating data that were within the normal range and 1 indicating an increase over the upper limit of the normal range. A logistic regression model was used to relate the probability of having an elevated γ-GT level. The area under the concentration-time curve (AUC) of VPA was used as the exposure variable. The AUC values were predicted from the population PK model. The probability, *Pr*, of having an elevated γ-GT level was expressed as the inverse logit function:
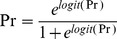
(1)in which the logit (Pr) is a linear function of the AUC:
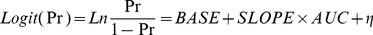
(2)where BASE represents the intercept, SLOPE is the slope relating the AUC to the effect and η is the individual random effect.

The logit (Pr) that described a nonlinear E_max_ relationship was also tested, and the equation is described below:
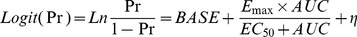
(3)in which E_max_ is the maximum increase in the probability of an elevated γ-GT level and EC_50_ is the exposure at which a half maximal increase in the probability is reached.

The influence of covariates on the logit (Pr) was systematically tested in the same manner as the PK analysis, except for using the equations described below:
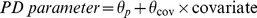
(4)where θ_p_ is the parameter estimate in an individual patient, while θ_cov_ is a fraction change in the PD parameter, i.e. logit (Pr), for each covariate group. The covariates tested were the age, gender, body weight, daily dose and duration of VPA therapy, *CYP2C9* genotype, *CYP2C19* genotype, *SOD2* genotype, *GSTM1* genotype, *GSTT1* genotype, complication with intellectual disability and co-administered antiepileptic drugs on the BASE, SLOPE, E_max_ and/or EC_50_. Typical values of the PD parameters were also considered at this step.

### Model Evaluation

A stratified nonparametric bootstrap analysis was performed to investigate the precision of the parameters of the population PK and PK-PD models implemented in the NONMEM and Wings for NONMEM programs (version 720; http://wfn.sourceforge.net/). One thousand replicated datasets were generated by random sampling with replacement, and were stratified according to the study population to ensure a representative study population distribution using the individual as the sampling unit. The population parameters of each dataset were subsequently estimated as described for the original estimation procedure.

For the population PK model, the goodness of fit was assessed by visually inspecting the scattered plots of the population model-predicted versus observed concentrations, the individually model-predicted versus observed concentrations and the conditional weighted residuals versus the population model-predicted concentrations.

A visual predictive check regarding the proportion of there being γ-GT elevation was also performed using 1,000 datasets that were randomly sampled from the original dataset, and was stratified by the statistically significant covariates identified in the final model. The visual predictive check is an internal validation tool that shows how well the model predicts the data on which the model was conditioned, and it compares the dependent variables derived from the original and the simulated datasets. [31]. The R-based software program, Xpose (version 4.4.1), was used for the graphical visualization of the results, and the PsN tool kit (version 3.5.3) was used for the post-processing of the results.

### Simulations of the PK-PD Parameters

Based on the final population PK-PD parameter estimates, the probability of there being γ-GT elevation were also simulated at the steady state in 1,000 individuals. The simulation processes were performed using the NONMEM program.

## Results

### Patient Demographics

237 patients fulfilled all of the inclusion criteria for the PK analysis and 169 fulfilled all of the inclusion criteria for the PK-PD analysis. The patient characteristics are presented in Table 1. The mean durations of follow-up were 3.2±4.0 and 6.6±5.1 years, respectively. Among the 237 patients included for the PK modeling, the allele frequencies of CYP2C9*3, CYP2C19*2 and CYP2C19*3 were 3.2%, 29.3% and 11.2%, respectively. The frequencies of the CYP2C9*1/*1, *1/*3 and *3/*3 genotypes were 93.7%, 6.3% and 0%, respectively, and those of *CYP2C19* homozygous EMs, heterozygous EMs and PMs were 35.9%, 47.2% and 16.9%, respectively. Among the 169 patients included for the PK-PD modeling, the frequency of the *SOD2* 16Ala allele was 12.7%, and the frequencies of the Val/Val, Val/Ala and Ala/Ala genotypes were 77.6%, 20.7% and 1.7%, respectively. The observed genotype frequency distributions for *CYP2C9*, *CYP2C19* and *SOD2* were consistent with the Hardy-Weinberg equilibrium (*P*>0.05). The number of patients with the *SOD2* Ala/Ala genotype (two patients among the 169 PK-PD modeling population) was too small to assess the effect of the genotype on the serum γ-GT elevation; therefore, the Ala/Ala and the Val/Ala genotypes were combined in the subsequent PK-PD analyses. The frequencies of the *GSTM1* null and *GSTT1* null genotypes were 56.8% and 47.9%, respectively, among the 169 patients included for the PK-PD modeling.

**Table 1:** A summary of the patient characteristics.

Patient Characteristics	PK analysis (N = 237)	PK-PD analysis (N = 169)	
	N (%) or mean ± SD (range)	N (%) or mean ± SD (range)	
Body weight [kg]	48.8±20.9 (9.6–120.5)	51.0±20.1 (13.0–120.5)	
Age [years]	17.2±8.3 (2.2–52.2)	18.0±7.8 (3.0–52.2)	
Gender (men/women)	137 (57.8%)/100 (42.2%)	102 (60.4%)/67 (39.6%)	
VPA dose [mg/day]	934.3±540.2 (100–2600)	903.8±502.7 (100–2600)	
VPA concentration [µg/mL]	68.15±26.54 (7.70–165.0)	67.8±25.7 (7.7–143.0)	
Seizure locus			
Generalize	111 (46.8%)	75 (44.4%)	
Partial	119 (50.2%)	87 (51.5%)	
Unidentified	7 (3.0%)	7 (4.1%)	
Seizure type			
Idiopathic	64 (27.0%)	42 (24.8%)	
Symptomatic	75 (31.7%)	50 (29.6%)	
Cryptogenic	98 (41.3%)	77 (45.6%)	
γ-GT [IU/L]	51.3±68.6 (7–515)	48.3±65.1 (2.4–515)	
ALT [IU/L]	19.1±15.7(5–134)	18.8±15.1 (4–134)	
AST [IU/L]	23.0±10.2 (9–103)	23.4±9.9 (9–103)	
Creatinine [mg/dL]	0.5±0.2 (0.1–1.5)	0.6±0.2 (0.1–1.5)	
BUN [mg/dL]	12.7±3.7 (3.5–28.3)	13.0±3.7 (3.5–28.3)	
Intellectual disability	131 (55.3%)	97 (57.4%)	
Monotherapy	378 (45.7%)	226 (47.8%)	
Co-administration			
CBZ	190 (23.0%)	94 (19.9%)	
CLB	128 (15.5%)	90 (19.0%)	
GBP	8 (1.0%)	5 (1.1%)	
PB	73 (8.8%)	28 (5.9%)	
PHT	88 (10.6%)	45 (9.5%)	
TPM	44 (5.3%)	29 (6.1%)	
ZNS	59 (7.1%)	27 (5.7%)	

PK = pharmacokinetic; PD = pharmacodynamic; N = number; SD = standard deviation; VPA = valproic acid; γ-GT: γ-glutamyltransferase; ALT: alanine aminotransferase; AST: aspartate aminotransferase; BUN = blood urea nitrogen; CBZ = carbamazepine; CLB = clobazam; GBP = gabapentine; PB = phenobarbital; PHT = phenytoin; TPM = topiramate; ZNS = zonisamide.

### Population PK Model

A total of 827 steady-state VPA concentrations were collected and made available for the PK analysis. The interval between the time of the last dose and the sampling time was distributed over 24 hours. A one-compartment model with exponential inter-individual variability on the *Ka*, *Vd/F*, *CL/F* and *ALAG* adequately described the data. The best residual error model was an additive model.

The daily VPA dose significantly influenced the *Vd/F* and *CL*/*F* of VPA. The covariates gender, co-administered carbamazepine (CBZ), phenobarbital (PB), phenytoin (PHT) and clobazam (CLB) significantly decreased the objective function values of the *CL*/*F* (Table S1). The *CYP2C9* and *CYP2C19* genotypes did not have any statistically significant influence on the *CL*/*F* in the forward-inclusion analysis (Table S1). Compared with the base model, the residual error was reduced by 17.9% in the final model. The final population pharmacokinetic model for VPA was as follows:
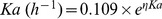
(5)

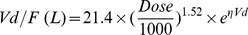
(6)

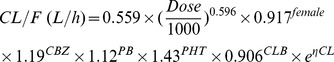
(7)

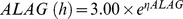
(8)where Dose is the daily VPA dose (mg/day); female = 1, male = 0; CBZ, PB, PHT or CLB = 1 if CBZ, PB, PHT or CLB was co-administered, and was otherwise 0; and η is the individual random effect.

Among 1,000 bootstrap runs, 965 runs exhibited successful minimization and were included in the bootstrap analysis. Table 2 shows the median parameter estimates obtained using the NONMEM program and the values with 95% confidence intervals (CIs) obtained using the bootstrap approach. The 95% CIs for all parameters obtained using the bootstrap approach was generally comparable to the estimates obtained using the NONMEM program (Table 2).

**Table 2:** The median values of the PK parameter estimates of VPA in the final population PK models obtained using the NONMEM program and the bootstrap analysis.

Parameter	NONMEM	Bootstrap Evaluation	
	Final Estimates	Median	95% CIs
*ALAG* (h)	3.00 (Fixed)	–	–
*Ka* (h^−1^)	0.109	0.104	0.0317–0.748
*Vd/F* (L)	21.4	21.2	8.01–68.1
*CL/F* (L/h)	0.559	0.558	0.520–0.589
Dose on *Vd/F* (L)	1.52	1.46	0.457–3.74
Dose on *CL/F* (L/h)	0.596	0.592	0.525–0.652
Gender on *CL/F* (L/h)	0.917	0.915	0.847–0.998
CBZ on *CL/F* (L/h)	1.19	1.20	1.13–1.29
CLB on *CL/F* (L/h)	0.906	0.907	0.852–0.970
PB on *CL/F* (L/h)	1.12	1.12	1.03–1.24
PHT on *CL/F* (L/h)	1.43	1.43	1.31–1.56
ω^2^ on *ALAG*	4.48×10^−9^	6.69×10^−5^	6.69×10^−5^–6.69×10^−5^
ω^2^ on *Ka*	7.77×10^−7^	8.82×10^−4^	8.81×10^−4^–8.82×10^−4^
ω^2^ on *Vd/F*	1.83×10^−7^	4.28×10^−4^	4.28×10^−4^–4.28×10^−4^
ω^2^ on *CL/F*	0.0587	0.241	0.202–0.281
σ^2^ (proportional error)	0.0617	0.247	0.224–0.268

VPA = valproic acid; PK = pharmacokinetic; NONMEM = nonlinear mixed-effect model; CIs = confidence intervals; *ALAG* = absorption lag time; *Ka* = absorption rate constant; *Vd/F* = volume of distribution; *CL/F* = apparent oral clearance; Dose = daily dose of VPA; CBZ = carbamazepine; CLB = clobazam; GBP = gabapentine; PB = phenobarbital; PHT = phenytoin; ω = coefficient of variation of inter-individual variability; σ = coefficient of variation of intra-individual variability; – = data not available.

A scatter plot of the population model-predicted and individually model-predicted concentrations versus the observed concentrations showed no bias, and the conditional weighted residuals were homogeneously distributed over the population model-predicted VPA concentrations (see Figure S1).

### PK-PD Model

A total of 472 γ-GT levels were collected and made available for the PK-PD analysis. The best fitted model for the probability of γ-GT elevation was a logistic regression model, in which the logit (Pr) was a linear function of the individual AUC value of VPA. During the forward-inclusion and the backward-elimination analyses, the *SOD2* Val/Val genotype, complication with intellectual disability and co-administered CBZ, PB and PHT were found to be significant covariates influencing the BASE of the logit (Pr) (Table S2). During the forward-inclusion analysis, the daily VPA dose, age, gender, body weight and duration of VPA therapy significantly influenced the SLOPE. During the backward-elimination analysis, the influence of the gender and duration of VPA therapy on the SLOPE were not statistically significant (Table S2), and therefore, these were removed from the full covariate model. The presence of an intellectual disability was associated with an increased number of co-administered CBZ, PB and PHT drugs (*P*<0.0001), whereas age, body weight and the daily VPA dose significantly correlated with each other (*P*<0.05). Therefore, we excluded the parameters of patient age, body weight and the use of co-administered CBZ, PB and PHT from the final model in order to increase the stability by reducing the degree of multicollinearity.

The final population PK-PD model for the probability of the γ-GT elevation was as follows:
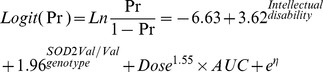
(9)where AUC is the individual AUC value of VPA that was simulated based on the population PK analysis; intellectual disability = 1 if an intellectual disability was present, and was otherwise 0; *SOD2* Val/Val genotype = 1, *SOD2* Val/Ala or Ala/Ala genotype = 0; and η is the individual random effect.

Among 1,000 bootstrap runs, 997 runs exhibited successful minimization and were included in the bootstrap analysis. Table 3 shows the median parameter estimates obtained using the NONMEM program and the values with 95% CIs obtained using the bootstrap approach. The 95% CIs for all parameters obtained using the bootstrap approach was generally comparable to the estimates obtained using the NONMEM program (Table 3).

**Table 3:** The median values of the PD parameter estimates of VPA in the final population PK-PD models obtained using the NONMEM program and the bootstrap analysis.

Parameter	NONMEM	Bootstrap Evaluation	
	Final Estimates (RSE, %)	Median	95% CIs
Base	−6.63 (17.5)	−6.68	−11.6–−4.82
Dose on SLOPE	1.55 (19.4)	1.56	0.83–2.29
Intellectual disability on BASE	3.62 (28.4)	3.62	1.87–8.94
*SOD2* genotype on BASE	1.96 (44.1)	2.02	0.33–4.42
ω^2^ on logit (Pr)	12.3 (43.4)	3.48	2.29–10.7

VPA = valproic acid; PD = pharmacodynamic; PK = pharmacokinetic; NONMEM = nonlinear mixed-effect model; RES = relative standard error; CIs = confidence intervals; Dose = daily dose of VPA; BASE = intercept; SLOPE = slope relating the AUC of VPA; SOD2 = superoxide dismutase 2; ω = coefficient of variation of inter-individual variability; logit (Pr) = logit function of probability of having an elevated γ-GT level.

**Figure 1:**
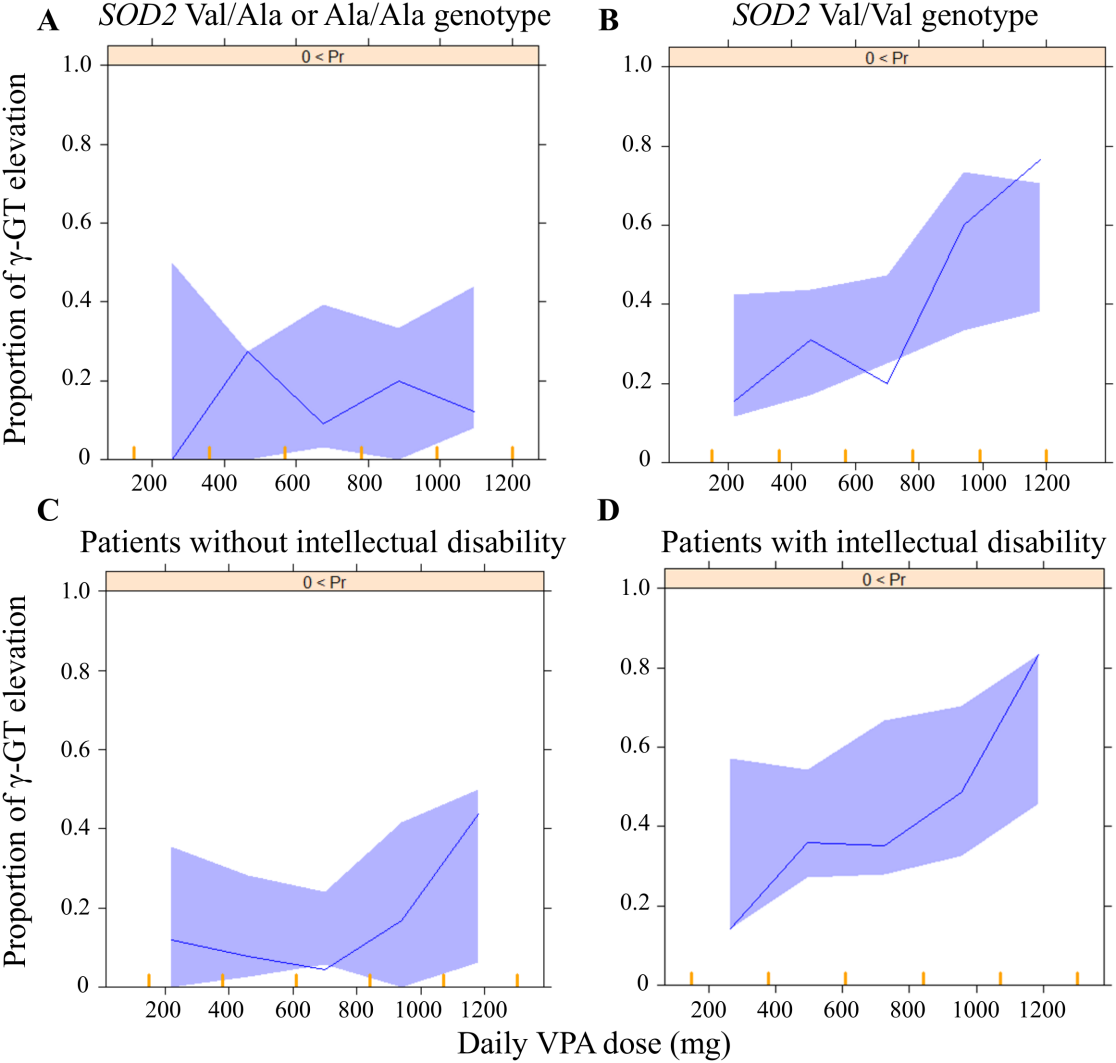
The visual predictive check of the PK-PD model in patients with the *SOD2* Ala/Ala or Val/Ala genotype (A), those with *SOD2* Val/Val genotype (B), those without intellectual disability (C) and those with intellectual disability (D), using the dataset of the study patients aged 30 years old or younger who were treated with 100 to 1,300 mg/day of VPA. The solid line represents the observed proportion of γ-GT elevation and the solid area represents the 90% prediction interval. PK = pharmacokinetic; PD = pharmacodynamics; SOD2 = superoxide dismutase 2; VPA = valproic acid γ-GT: γ-glutamyltransferase.

A visual predictive check regarding the proportion of γ-GT elevation using 1,000 datasets according to the *SOD2* genotype and complication with intellectual disability is shown in Figure 1. In this study, we included patients 2 to 52 years of age among the original population in the PK-PD analysis (Table 1); however, 93.3% of the patients were 30 years of age or younger (see Figure S2). Therefore, for the visual predictive assessment, we selected patients 30 years of age or younger treated with 100 to 1,300 mg/day of VPA (the usual dose in Japan) in order to predict the data for our PK-PD model appropriately by reducing the variability among the patient ages in the simulated 1,000 datasets. The visual predictive check indicated that the final parameter estimates were reliable (Figure 1).

When 800 mg/day of VPA was administered to patients without any co-treatment, the predicted mean percentages of the subjects with γ-GT elevation were 9.4%, 20.2%, 31.6% and 52.6%, and the mean odds ratios (95% CIs) for the γ-GT elevation during VPA therapy were 1 [reference value], 2.44 (1.88–3.17), 4.47 (3.48–5.74) and 10.74 (8.30–13.75) in patients with the *SOD2* Val/Ala or Ala/Ala genotype without intellectual disability, those with the *SOD2* Val/Val genotype without intellectual disability, those with the *SOD2* Val/Ala or Ala/Ala genotype and intellectual disability and those with the *SOD2* Val/Val genotype and intellectual disability, respectively (Table 4). The predicted mean percentages of the subjects with γ-GT elevation were about 2- to 3-fold, 3- to 4-fold and 4- to 8-fold higher in the patients with the *SOD2* Val/Val genotype without intellectual disability, those with the *SOD2* Val/Ala or Ala/Ala genotype and intellectual disability and those with the *SOD2* Val/Val genotype and intellectual disability, respectively, than in those with the *SOD2* Val/Ala or Ala/Ala genotype without intellectual disability (Table 4).

**Table 4:** The predicted mean percentages of the subjects with γ-GT elevation and the mean odds ratios (95% CIs) for γ-GT elevation during VPA therapy according to the *SOD2* Val16Ala genotype and complication with intellectual disability when different daily doses of VPA were administered to patients without any co-treatment.

Dose (mg)	Intellectualdisability	*SOD2* genotype	γ-GTelevation (%)	Odds ratio (95% CIs)
400	–	Val/Ala or Ala/Ala	5.8	1
	–	Val/Val	13.0	2.42 (1.75–3.34)
	+	Val/Ala or Ala/Ala	24.9	5.38 (3.98–7.27)
	+	Val/Val	42.4	11.91 (8.89–15.98)
500	–	Val/Ala or Ala/Ala	6.0	1
	–	Val/Val	14.1	2.56 (1.87–3.51)
	+	Val/Ala or Ala/Ala	25.5	5.32 (3.95–7.16)
	+	Val/Val	43.8	12.12 (9.08–16.17)
600	–	Val/Ala or Ala/Ala	5.2	1
	–	Val/Val	16.9	3.67 (2.66–5.07)
	+	Val/Ala or Ala/Ala	27.8	6.96 (5.10–9.49)
	+	Val/Val	44.8	14.68 (10.82–19.91)
700	–	Val/Ala or Ala/Ala	7.7	1
	–	Val/Val	17.2	2.50 (1.88–3.32)
	+	Val/Ala or Ala/Ala	30.6	5.30 (4.05–6.94)
	+	Val/Val	48.1	11.17 (8.58–14.54)
800	–	Val/Ala or Ala/Ala	9.4	1
	–	Val/Val	20.2	2.44 (1.88–3.17)
	+	Val/Ala or Ala/Ala	31.6	4.47 (3.48–5.74)
	+	Val/Val	52.6	10.74 (8.30–13.75)
900	–	Val/Ala or Ala/Ala	9.6	1
	–	Val/Val	21.1	2.52 (1.94–3.27)
	+	Val/Ala or Ala/Ala	34.9	5.04 (3.94–6.46)
	+	Val/Val	55.6	11.77 (9.22–15.03)
1000	–	Val/Ala or Ala/Ala	10.8	1
	–	Val/Val	24.2	2.65 (2.07–3.39)
	+	Val/Ala or Ala/Ala	35.6	4.57 (3.60–5.80)
	+	Val/Val	56.2	10.63 (8.40–13.46)
1100	–	Val/Ala or Ala/Ala	13.1	1
	–	Val/Val	27.8	2.55 (2.02–3.20)
	+	Val/Ala or Ala/Ala	42.6	4.90 (3.93–6.13)
	+	Val/Val	57.8	9.05 (7.24–11.30)
1200	–	Val/Ala or Ala/Ala	14.8	1
	–	Val/Val	28.1	2.25 (1.80–2.81)
	+	Val/Ala or Ala/Ala	46.6	5.01 (4.04–6.20)
	+	Val/Val	65.3	10.83 (8.71–13.46)

γ-GT: γ-glutamyltransferase; VPA = valproic acid; Dose = daily dose of VPA; SOD2 = superoxide dismutase 2; CIs = confidence intervals; − = absent; + = present.

## Discussion

**Figure 2:**
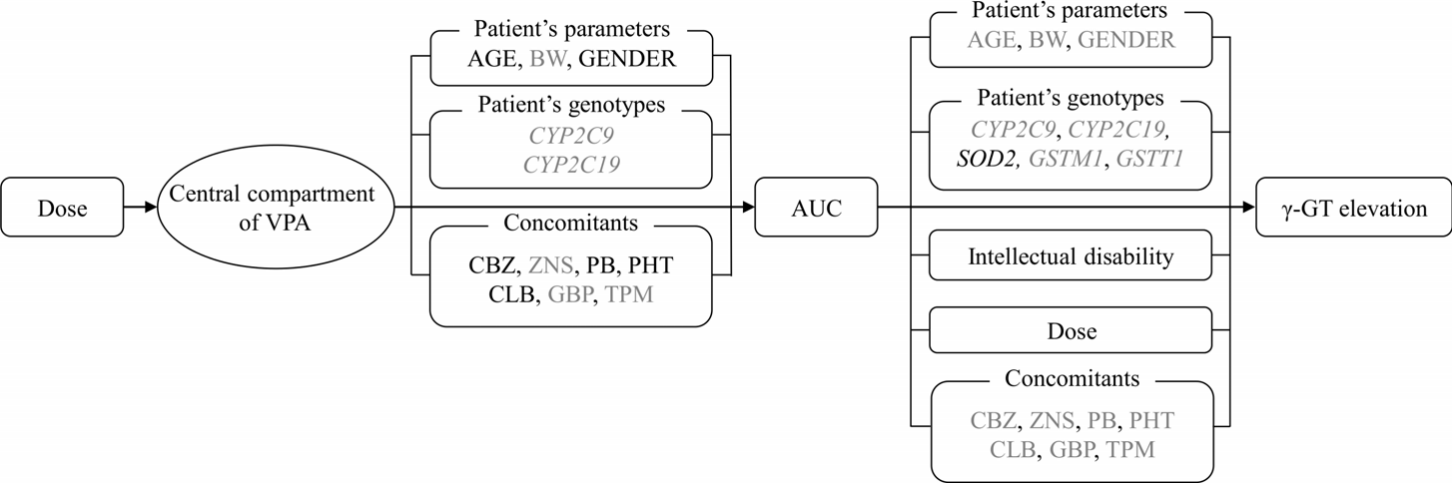
The relationships evaluated in the framework for the γ-GT elevation during VPA therapy. The solid lines indicate the relationships included in the final population PK and PK-PD models. Black letters indicate relationships included in the final models, and grey letters indicate relationships investigated, but not included, in the final models. PK = pharmacokinetic; PD = pharmacodynamics; VPA = valproic acid γ-GT: γ-glutamyltransferase.

In this study, we developed an equation to describe the relationship between the serum VPA concentrations and the risk of γ-GT elevation by developing population PK and PK-PD models. During the model developments, we evaluated the associations of the VPA-induced γ-GT elevation with patient-specific covariates, including the *SOD2*, *GSTM1* and *GSTT1* genotypes, in Japanese patients with epilepsy (see Figure 2). A one-compartment model with exponential inter-individual variability of the PK parameters adequately described the data; while a logistic model was successfully applied to describe the VPA-induced γ-GT elevation, in which the logit function of probability was a linear function of the VPA exposure (Tables 2 and 3). The results of our PK-PD model indicated that the risk of γ-GT elevation was significantly related to the VPA exposure.

γ-GT is an established liver function test parameter used to determine whether there has been alcohol-associated toxicity [1]. It has also been reported to be a strong independent biomarker for metabolic syndrome, cardiovascular diseases and metabolic diseases, as well as the all-cause mortality [2], [7], [8]. Recent studies suggested that the γ-GT level is a significant predictor of NAFLD [3], [4]. Meanwhile, the potential metabolic abnormalities and hepatotoxicity of VPA are of major concern [9]. Long-term VPA therapy is associated with a high prevalence of NAFLD in adolescents (36.0%) [12] and adults (60.9%) [11] with epilepsy.

Since VPA is a fatty acid that can be metabolized through endogenous pathways in the mitochondria [24], mitochondrial dysfunction has been implicated in the pathogenesis of VPA-induced hepatotoxicity [9], [13]. SOD2 represents the first line of cellular defense against mitochondrial reactive oxygen species [14]. In our previous case-control study, we found that the *SOD2* Val16Ala polymorphism was associated with the risk of a mild increase in the γ-GT level in VPA-treated patients [16]. The findings of the present study demonstrated that the *SOD2* Val/Val genotype could contribute to the relationship between VPA exposure and γ-GT elevation during long-term VPA therapy. Based on the results of this study, the dose adjustment of VPA should be performed to reduce the risk of γ-GT elevation, especially in patients with the *SOD2* Val/Val genotype (Figure 1 and Table 4).

The *GSTM1* null and *GSTT1* null genotypes have been reported to be associated with the risk of cardiovascular or metabolic diseases, including NAFLD, in the general population [18], [19]. A previous case-control study reported that the *GSTM1* null and *GSTT1* null genotypes were associated with elevation of the γ-GT level in VPA-treated patients [21]. However, we did not identify these polymorphisms as statistically significant covariates in our PK-PD model (Table S2). The findings of this study may therefore indicate that the *GSTM1* null or *GSTT1* null genotype does not play a major independent role in the VPA-induced γ-GT elevation.

The association between epilepsy and intellectual disability has been well described in the literature [23]. A population-based retrospective cohort study showed that the risk of developing intellectual disability was high in patients with epilepsy (hazard ratio: 31.5, 95% CIs: 18.9 to 52.4) [23]. In this study, intellectual disability was also identified as a statistically significant covariate for γ-GT elevation in the VPA-treated patients (Figure 2 and equation 9). In our study subjects, the complication with intellectual disability was associated with the increased numbers of co-administered CBZ, PB and PHT (*P*<0.0001). These enzyme-inducing antiepileptic drugs are known to increase the γ-GT levels [9], [32]. A previous study indicated that VPA-induced γ-GT elevation was found in patients with polytherapy, but not in those with monotherapy [29]. Meanwhile, individuals with intellectual disabilities have also been reported to have higher levels of overweight and obesity than those without [33]. Therefore, we speculated that the patients with intellectual disability may be more likely to be co-administered with enzyme-inducing antiepileptic drugs and/or to have a weight gain, thus resulting in the γ-GT elevation, although the underlying mechanism remains unknown. Nevertheless, the findings of this study may suggest that patients with intellectual disability should be carefully monitored for VPA-induced γ-GT elevation (Figure 1 and Table 4).

VPA is highly protein bound (87–95%), thus resulting in low clearance [24]. There are at least three routes of VPA metabolism in humans: glucuronidation and β-oxidation in the mitochondria (both considered to be major routes accounting for 50% and 40% of the dose, respectively), and CYP-, such as CYP2C9 and CYP2C19, mediated-oxidation in the liver (considered to be a minor route, accounting for ∼10% of the dose) [24]. Similar to previous published population PK models for VPA [34]–[36], in the present study, a one-compartment model adequately described the VPA concentrations (Figure S1).

In this study, we could not find any influence of the *CYP2C9* and *CYP2C19* polymorphisms on the PK parameters of VPA. These results are in line with a recent Iranian study, showing that no association was observed between the CYP2C9*3 genotype and plasma VPA concentrations [37]. However, two other studies demonstrated an association between the *CYP2C9* or *CYP2C19* genotype and the PK parameters of VPA. Jiang et al. reported that the *CYP2C9* and *CYP2C19* genotypes significantly influenced the population PK parameters of VPA in Chinese patients with epilepsy [34]. Tan et al. indicated that subjects who were CYP2C9*3 allele carriers had higher mean plasma VPA concentrations than the non-carriers [38]. The discrepancy between the findings may be attributed to the sample sizes, ages, co-administered antiepileptic drug(s) or races evaluated in the studies. On the other hand, female gender, CBZ, PB, PHT and CLB were identified as statistically significant PK covariates in this study. There is evidence for females having lower UDP-glucuronosyltransferases (UGTs) activity [39]. CBZ, PB and PHT are all inducers of CYP2C9, CYP2C19 and UGTs [40]. Several population PK studies indicated that female gender decreases the clearance of VPA, whereas co-treatments with enzyme-inducing antiepileptic drugs increase it [35], [36]. Additionally, one previous study of pediatric patients reported that CLB reduced the apparent clearance of VPA [41], although the underlying mechanism remains unknown. Therefore, our PK parameter estimates of VPA might support these previous findings.

A major limitation of this study was the retrospective study design that investigated a small number of patients, which may have resulted in a lack of power. First, in this study, we could not determine the standard errors of the PK parameters in the final PK model developed (Table 2). This finding may be due to the complexity of our PK models incorporating many covariates (i.e. age, body weight, genotypes and co-administered drugs) using a limited number of samples per individual and the routine therapeutic drug monitoring data (i.e. sparse sampling). Second, we could not include the influence of several statistically significant covariates, such as enzyme-inducing antiepileptic drugs (Table S2), on the risk of γ-GT elevation in our final PK-PD model in order to increase the model stability. Third, since we included patients 2 to 52 years of age (Table 1 and Figure S2), further studies with larger numbers of patients are needed to verify the influence of the patient age on the findings of the present study. Fourth, possible effects of other polymorphisms in the genes involved in the disposition of VPA, such as *UGTs* [24] and those involved in the VPA-induced liver dysfunction, such as *PLOG* [9], [24], also cannot be ruled out. Lastly, we included only Japanese subjects; therefore, it is unclear whether our results can be generalized to other populations, such as white or black populations. The current findings are thus needed to be verified with a larger number of patients, including those from other ethnic groups. Nevertheless, our population PK results of the bootstrap analysis showed that the median parameter estimates obtained from 965 bootstrap data sets were generally comparable with the estimates obtained using the NONMEM program (Table 2), whereas the goodness of fit suggests that our PK model adequately described the original data (Figure S1). Furthermore, the PK-PD results of the bootstrap analysis also showed that the median parameter estimates obtained from 997 bootstrap data sets were generally comparable with the estimates obtained using the NONMEM program (Table 3), and the visual predictive check among 1,000 datasets indicated that the final parameter estimates were reliable (Figure 1).

In conclusion, this study demonstrated that the *SOD2* Val16Ala polymorphism has an impact on the relationship between VPA exposure and γ-GT elevation in patients with epilepsy, as determined using a population PK-PD modeling approach. Since patients with epilepsy usually receive long-term VPA from childhood, our results suggest that VPA-treated patients should be carefully monitored for γ-GT elevation, especially those with the *SOD2* Val/Val genotype. Furthermore, the findings of this study indicate that determining the *SOD2* genotype may be helpful for preventing the VPA-induced γ-GT elevation.

## Supporting Information

Figure S1 **The goodness of fit of the final population PK model.** The population-predicted (A) and individually-predicted (B) versus observed VPA concentrations in Japanese patients with epilepsy. The conditional weighted residuals versus population-predicted VPA concentrations in patients with epilepsy (C).
(TIF)

Figure S2 **The frequency distribution of the patient age for the datasets in the PK-PD analysis.**
(TIF)

Table S1 The effects of the tested covariates on the objective function of the PK parameters of VPA.
(DOCX)

Table S2 The effects of the tested covariates on the objective function of the PK-PD parameters regarding the probability of a VPA-induced γ-GT elevation.
(DOCX)
