# Supplementary figures and images for: Impact of the Superoxide Dismutase 2 Val16Ala Polymorphism on the Relationship between Valproic Acid Exposure and Elevation of γ-Glutamyltransferase in Patients with Epilepsy: A Population Pharmacokinetic-Pharmacodynamic Analysis
Source: PLoS One. 2014 Nov 5;9(11):e111066. doi: 10.1371/journal.pone.0111066 (PMC4220988; doi:10.1371/journal.pone.0111066)

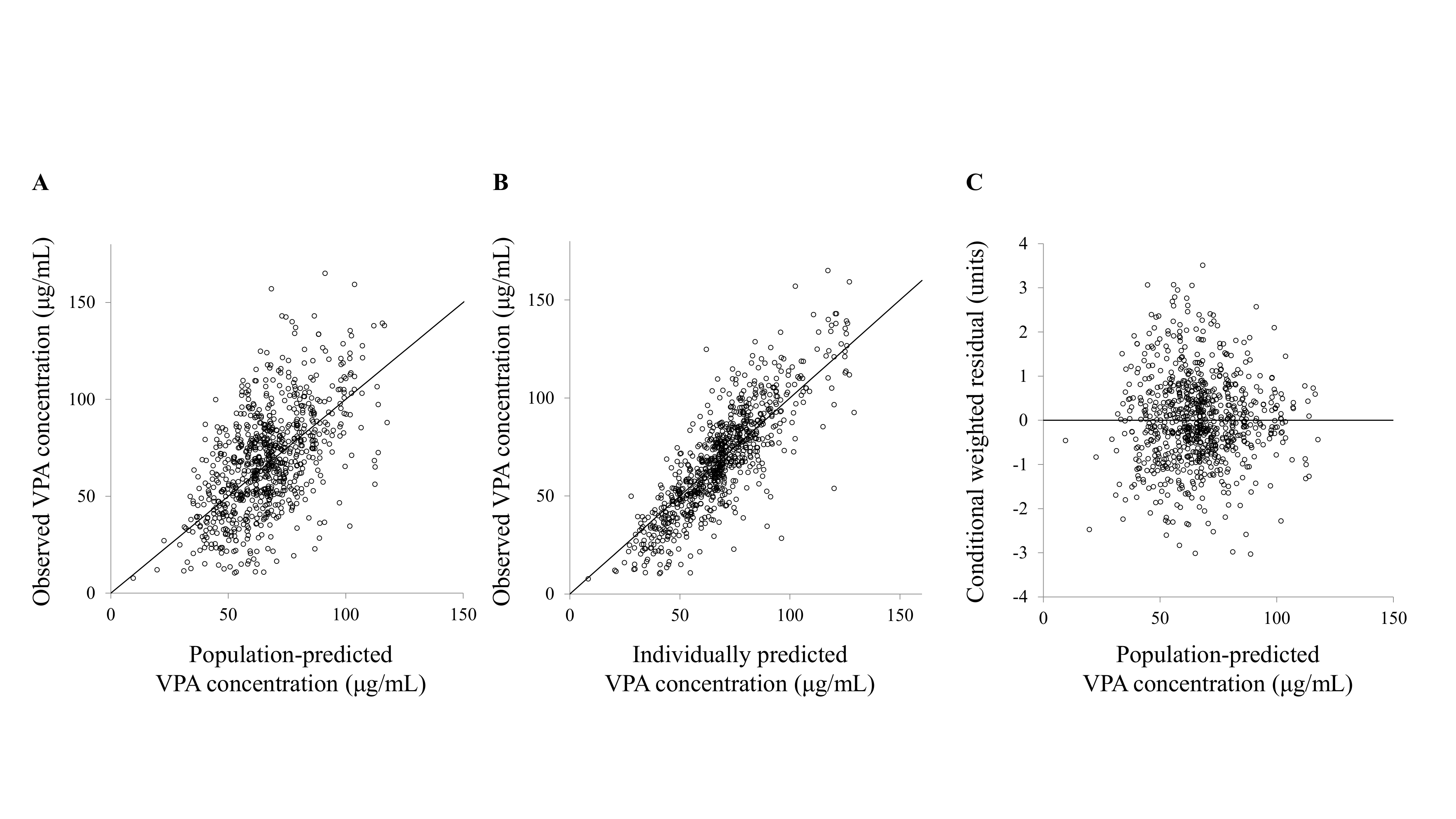

Supplement: Figure S1 — The goodness of fit of the final population PK model. The population-predicted (A) and individually-predicted (B) versus observed VPA concentrations in Japanese patients with epilepsy. The conditional weighted residuals versus population-predicted VPA concentrations in patients with epilepsy (C). (TIF) [file pone.0111066.s001.tif]
