# Supplementary material for: Impact of the Superoxide Dismutase 2 Val16Ala Polymorphism on the Relationship between Valproic Acid Exposure and Elevation of γ-Glutamyltransferase in Patients with Epilepsy: A Population Pharmacokinetic-Pharmacodynamic Analysis
Source: PLoS One. 2014 Nov 5;9(11):e111066. doi: 10.1371/journal.pone.0111066 (PMC4220988; doi:10.1371/journal.pone.0111066)
Supplement: Table S1 — The effects of the tested covariates on the objective function of the PK parameters of VPA. (DOCX) [file pone.0111066.s003.docx]

**Table S1**

| PK Parameter | Tested Covariate | Forward inclusion step | Backward elimination step |
| --- | --- | --- | --- |
|  |  | *P* value | *P* value |
| *Vd/F* | Body weight | ≥0.05 | ─ |
|  | VPA dose | <0.05 | <0.05 |
| *CL/F* | Age | <0.05 | ─ |
|  | Gender | <0.05 | <0.05 |
|  | Body weight | <0.05 | ─ |
|  | VPA dose | <0.05 | <0.05 |
|  | *CYP2C9* genotypes | ≥0.05 | ─ |
|  | *CYP2C19* genotypes | ≥0.05 | ─ |
|  | Co-administered AED |  |  |
|  | CBZ | <0.05 | <0.05 |
|  | CLB | <0.05 | <0.05 |
|  | GBP | ≥0.05 | ─ |
|  | PB | <0.05 | <0.05 |
|  | PHT | <0.05 | <0.05 |
|  | TPM | ≥0.05 | ─ |
|  | ZNS | ≥0.05 | ─ |

PK = pharmacokinetic; VPA = valproic acid; *Vd/F* = volume of distribution; *CL/F* = apparent oral clearance; CYP = cytochrome P450; AED = antiepileptic drug; CBZ = carbamazepine; CLB = clobazam; GBP = gabapentine; PB = phenobarbital; PHT = phenytoin; TPM = topiramate; ZNS = zonisamide; ─ = data not available.
