# Supplementary material for: Impact of the Superoxide Dismutase 2 Val16Ala Polymorphism on the Relationship between Valproic Acid Exposure and Elevation of γ-Glutamyltransferase in Patients with Epilepsy: A Population Pharmacokinetic-Pharmacodynamic Analysis
Source: PLoS One. 2014 Nov 5;9(11):e111066. doi: 10.1371/journal.pone.0111066 (PMC4220988; doi:10.1371/journal.pone.0111066)
Supplement: Table S2 — The effects of the tested covariates on the objective function of the PK-PD parameters regarding the probability of a VPA-induced γ-GT elevation. (DOCX) [file pone.0111066.s004.docx]

**Table S2**

| PD Parameter | Tested Covariate | Forward inclusion step | Backward elimination step |
| --- | --- | --- | --- |
|  |  | *P* value | *P* value |
| BASE | Age | ≥0.05 | ─ |
|  | Body weight | ≥0.05 | ─ |
|  | Gender | ≥0.05 | ─ |
|  | VPA dose | ≥0.05 | ─ |
|  | Duration of VPA therapy | ≥0.05 | ─ |
|  | *CYP2C9* genotypes | ≥0.05 | ─ |
|  | *CYP2C19* genotypes | ≥0.05 | ─ |
|  | *SOD2* genotypes | <0.05 | <0.05 |
|  | *GSTM1* genotypes | ≥0.05 | ─ |
|  | *GSTT1* genotypes | ≥0.05 | ─ |
|  | Intellectual disability | <0.05 | <0.05 |
|  | Co-administered AED |  |  |
|  | CBZ | <0.05 | <0.05^1^ |
|  | CLB | ≥0.05 | ─ |
|  | GBP | ≥0.05 | ─ |
|  | PB | <0.05 | <0.05^1^ |
|  | PHT | <0.05 | <0.05^1^ |
|  | TPM | ≥0.05 | ─ |
|  | ZNS | ≥0.05 | ─ |
| SLOPE | Age | <0.05 | <0.05^1^ |
|  | Body weight | <0.05 | <0.05^1^ |
|  | Gender | <0.05 | ≥0.05 |
|  | VPA dose | <0.05 | <0.05 |
|  | Duration of VPA therapy | <0.05 | ≥0.05 |
|  | *CYP2C9* genotypes | ≥0.05 | ─ |
|  | *CYP2C19* genotypes | ≥0.05 | ─ |
|  | *SOD2* genotypes | ≥0.05 | ─ |
|  | *GSTM1* genotypes | ≥0.05 | ─ |
|  | *GSTT1* genotypes | ≥0.05 | ─ |
|  | Intellectual disability | ≥0.05 | ─ |
|  | Co-administered AED |  |  |
|  | CBZ | ≥0.05 | ─ |
|  | CLB | ≥0.05 | ─ |
|  | GBP | ≥0.05 | ─ |
|  | PB | ≥0.05 | ─ |
|  | PHT | ≥0.05 | ─ |
|  | TPM | ≥0.05 | ─ |
|  | ZNS | ≥0.05 | ─ |

^1^ These covariates were removed from the final model in order to increase the stability by reducing the degree of multicollinearity.

PK = pharmacokinetic; PD = pharmacodynamic; VPA = valproic acid; γ-GT = γ-glutamyltransferase; BASE = intercept; SLOPE = slope relating the area under the concentration-time curve of VPA; SOD2 = superoxide dismutase 2; CYP = cytochrome P450; GST = glutathione *S*-transferase; AED = antiepileptic drug; CBZ = carbamazepine; CLB = clobazam; GBP = gabapentine; PB = phenobarbital; PHT = phenytoin; TPM = topiramate; ZNS = zonisamide; ─ = data not available.
